# Relationship between different diet indices and frailty and mortality in population with CKD

**DOI:** 10.3389/fnut.2025.1602587

**Published:** 2025-10-07

**Authors:** Jing Peng, Yuhan He, Bohua Zhang, Ruoxi Liao, Baihai Su

**Affiliations:** ^1^Department of Nephrology, West China Hospital, Sichuan University, Chengdu, China; ^2^Med+ Biomaterial Institute, West China Hospital, Sichuan University, Chengdu, China

**Keywords:** diet, dietary indices, frailty, chronic kidney disease, mortality

## Abstract

**Background:**

Modification of diet is a convenient and cost-effective approach proven to be beneficial for populations with chronic kidney disease (CKD). Nutritional status is closely related to the frailty status, and both are associated with health outcomes. However, in populations with CKD, the prognostic value of different dietary indices for survival and how frailty will influence their association remains unclear. The objectives of our analysis were: (1) to assess the associations between frailty and seven dietary indices in the population with CKD; (2) to evaluate the mortality risk of frailty and different dietary scores in CKD; (3) to explore the association between dietary scores and mortality after adjustment for the frailty index.

**Methods:**

A total of 4,445 participants with CKD (aged ≥ 20 years) from the 2007–2016 cohorts of the National Health and Nutrition Examination Survey (NHANES) were enrolled. Nutrition Index (NI), Dietary Inflammatory Index (DII), Healthy Eating Index-2020 (HEI-2020), Mediterranean Diet Score (MED), Dietary Approaches to Stop Hypertension (DASH), Dietary Acid Load (DAL), and Composite Dietary Antioxidant Index (CDAI) were calculated based on dietary intake information from the first 24-h recall data. Linear regression models were performed to evaluate the association between different dietary scores and the frailty index (FI). Cox regression models were utilized to identify the associations of dietary indices and frailty with mortality.

**Results:**

FI was significantly higher in participants with CKD compared to the overall population. There was a significant relationship between DII, NI, CDAI, HEI-2020, and MED scores with frailty in CKD patients. Frailty index, DII, NI, and HEI-2020 scores were significantly associated with increased mortality risk in individuals with CKD. The relationship between DII score, NI score, HEI-2020 score, and mortality changed when adjusting for frailty.

**Conclusion:**

In individuals with CKD, frailty was associated with DII, NI, CDAI, HEI-2020, and MED scores. A higher FI was significantly associated with increased risk of all-cause mortality. Additionally, higher DII, NI, and lower HEI-2020 scores were related to mortality risk. After adjustment for FI, only a higher NI score (3-year and 5-year mortality) and a lower HEI-2020 score (3-year and 8-year mortality) were associated with higher mortality risk.

## 1 Introduction

Chronic kidney disease (CKD) is a major public health concern and is expected to become the 5th leading cause of death by 2040 (1). CKD is a progressive disease characterized by sustained damage to the structure or function of the kidney for more than 3 months, with limited treatment options and high morbidity and mortality (2). The management of CKD has been focused on medications and dialysis, while non-pharmacological strategies are usually ignored (3). Traditional low-sodium, low-fat, and low-protein diets are recognized as beneficial for patients with CKD, especially in patients with CKD stage 3–5 (4, 5), but these diets only focus on individual nutrients irrespective of the balance between different dietary components.

Dietary patterns are combinations of various foods that take into account food kinds and quantities, which may serve as better methods to assess the relationship between diet and risk of health outcomes (6, 7). The adherence to specific dietary patterns can be evaluated by corresponding dietary scores. Common dietary scores studied in chronic diseases include the Mediterranean Diet Score (MED), the Healthy Eating Index (HEI), and the Dietary Approaches to Stop Hypertension (DASH) (8). Adherence to a Mediterranean diet could reduce the risk of all-cause and cardiovascular mortality, incidence of certain cancers, and neurological diseases (9). The DASH diet was originally designed to help control high blood pressure, but has also been associated with other health outcomes, including cognitive function and cardiovascular disease mortality (10, 11). The HEI is an index measuring alignment with the Dietary Guidelines for Americans. Previous studies revealed the significant associations between HEI-2015 and all-cause and cause-specific mortality (12). Other metrics derived from nutrients or food or food groups, such as Nutrition Index (NI), Dietary Inflammatory Index (DII), Dietary Acid Load (DAL), and Composite Dietary Antioxidant Index (CDAI), are also related to health outcomes (13–16).

Different dietary indices focused on different aspects of individual diet and their influence on health outcomes might vary between the general population and individuals with diseases. Impairment of renal function is an important characteristic in CKD, thus causing electrolyte disturbance, metabolic acidosis, and microinflammation (3). Consequently, it is unclear whether these conclusions apply to populations with CKD as well.

Recent studies revealed that frailty was associated with several different dietary indices and may influence the mortality risk of these dietary scores in adults across a wide age spectrum (17, 18). Frailty was common in CKD and was associated with a higher risk of cardiovascular events, end-stage kidney disease, and mortality (19–21). According to the study by Wilkinson et al. (21), 75.3% of those with CKD were frail. Similarly, Hannan et al. (19) reported that only 37% of individuals with CKD were non-frail. The underlying mechanisms of the high prevalence of frailty in CKD remain unclear. Inflammation, oxidative stress, and malnutrition may play important roles (22). However, limited research has explored the relationship of frailty with different dietary patterns or indices and the mortality risk of diet-related scores after adjustment for FI in populations with CKD.

Therefore, based on the data from National Health and Nutrition Examination Survey (NHANES) program, the present study aimed to (1) assess the associations between frailty with seven dietary indices in population with CKD; (2) evaluate the mortality risk of frailty and these dietary scores in CKD; (3) explore the association between dietary scores and mortality after adjustment for frailty index.

## 2 Materials and methods

### 2.1 Study population

All data used in this analysis were based on the National Health and Nutrition Examination Survey (NHANES) program. NHANES is a series of continued surveys assessing the health and nutritional status of people in the United States. Publicly available data in NHANES include demographic, dietary, examination, laboratory, and questionnaire data, released in two-year cycles (23). This study included 23,683 participants aged ≥ 20 years from the 2007–2016 survey cycles. We identified 4,640 participants with CKD and complete dietary data.

CKD was defined as estimated glomerular filtration rate (eGFR) < 60 mL/min/1.73 m^2^ or urine albumin-creatinine ratio (UACR) ≥ 30 mg/g. The CKD-Epidemiology Collaboration (EPI) equation is used to estimate the eGFR. The stage of CKD was based on eGFR according to the Kidney Disease: Improving Global Outcomes (KDIGO) 2024 guideline (24).

UACR (mg/g) = urinary albumin level (mg/dL)/urinary creatinine level (g/dL).

**Figure 1:**
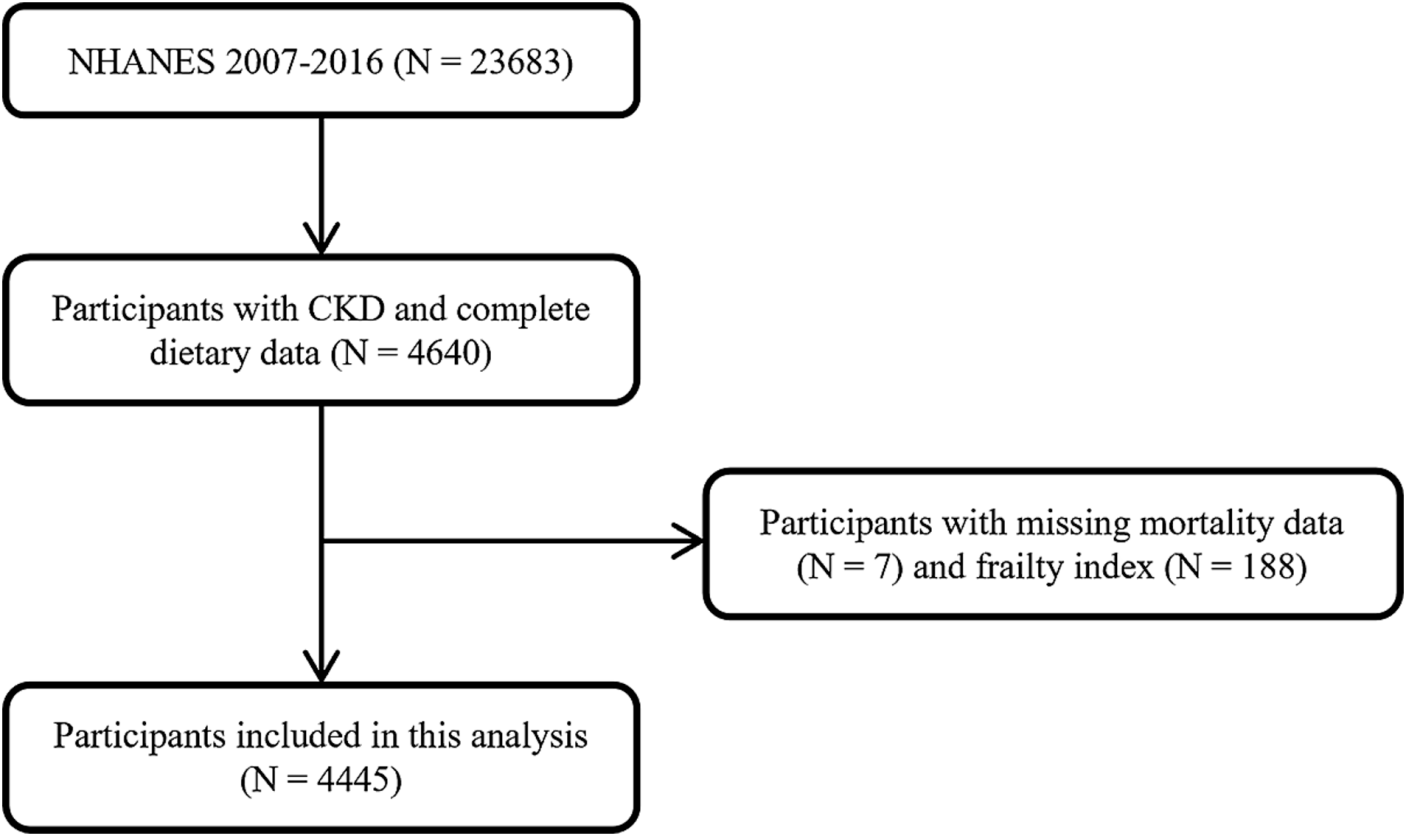
Flowchart of the study population screening.

After exclusion of 195 participants with missing mortality data (*N* = 7) and frailty index (*N* = 188), 4,445 participants were finally included in this analysis (Figure 1).

Mortality follow-up data were obtained from the National Death Index from the date of survey participation through 31 December 2019 (25). The follow-up time was calculated from the mobile examination center date to the date of death or the end of the mortality follow-up period. All participants provided written informed consent approved by the National Center for Health Statistics.

### 2.2 Collection of nutrition-related data

Detailed dietary intake information was obtained from the first 24-h recall data collected in-person by experienced interviewers in the Mobile Examination Center of NHANES. To calculate the dietary scores, corresponding data from the Food Patterns Equivalents Database files were also collected. All diet-related variables used to calculate the dietary scores are listed in Supplementary Table S1.

#### 2.2.1 Nutrition Index

The Nutrition Index (NI) is a newly developed score by constructing nutrition-related parameters from NHANES that were related to higher frailty with a deficit accumulation approach (15). Parameters used to calculate NI included 18 dietary variables (energy, energy per weight, protein, protein per weight, carbohydrate, percentage of saturated fat, vitamins A, C, B1, B2, B3, and B6, folate, phosphorous, copper, sodium, selenium, fish oil), 3 anthropometric measurements (body mass index, body weight change in the past year, waist circumference), and 10 laboratory tests (lymphocyte count, hemoglobin, mean corpuscular volume and serum albumin, vitamin D, iron, creatinine, triglyceride, high density lipoprotein (HDL)-cholesterol, and glucose) (18). Each variable was scored “0” if the value was in the normal range and “1” otherwise (Supplementary Table S2). Scores for each variable were summed up and divided by 31 to obtain the NI score. NI is a value ranging between 0 and 1, with a higher score indicating worse nutritional status. Individuals with > 20% missing variables were excluded from the analysis.

#### 2.2.2 Dietary Inflammatory Index

The dietary Inflammatory Index (DII) is a literature-derived, population-based score to assess the inflammatory potential of diets applying to different dietary databases (26). A total of 45 dietary components with inflammatory potential were used for DII calculation. In our study, 28 nutrients were available in the first 24-h recall data of NHANES (Supplementary Table S1). The calculation of DII was based on the standard protocol (26). In brief, the intake of individual dietary components was normalized and converted to a percentile score using the corresponding mean value and standard deviation. This value was then multiplied by its respective inflammatory effect score and summed to obtain the final DII score. Higher DII scores represent more pro-inflammatory diets. The detailed components and calculations for DII are listed in Supplementary Table S3.

#### 2.2.3 Healthy Eating Index-2020

The Healthy Eating Index (HEI) was first released by the United States Department of Agriculture’s (USDA) Center and was revised in 2005. The HEI was updated every 5 years, and the HEI-2020 is the latest iteration of the index. HEI-2020 contains 13 components, and the total score is the sum of the scores of adequacy components and moderation components (27). The detailed components and scoring standards of the HEI-2020 are listed in Supplementary Table S4. The maximum score is 100 points, and higher scores indicate better diet quality.

#### 2.2.4 Mediterranean diet score

The Mediterranean diet was globally acknowledged as a healthy dietary pattern that can reduce the risk of cardiovascular disease, certain cancers, and mortality (9, 28). Here, a total of nine components were included in the calculation of the MED score (vegetables, fruits, nuts, whole grains, legumes, fish, ratio of monounsaturated to saturated fat, red and processed meats, and alcohol). Individuals with intake above/below the median intake received 1/0 point for each component, while 1 point was given when the consumption of red and processed meat exceeded the median (29). The final score ranges from 0 to 9, with a higher score representing better adherence to the Mediterranean diet. The detailed calculation for the MED score is shown in Supplementary Table S5.

#### 2.2.5 Dietary Approaches to Stop Hypertension

The Dietary Approaches to Stop Hypertension (DASH) diet was first proposed for the control of blood pressure (30). It refers to a diet rich in fruits, vegetables, and low-fat dairy foods with reduced fat. Nine components were used for constructing the DASH score. One point was assigned when the intake of an individual component meets the goal of the DASH diet. If the intake meets an intermediate goal, the following formula was used to calculate the point. Zero point was given when the intake met neither goal.

$$\mathrm{Point}=\left(\begin{array}{l} \mathrm{actual intake}\\ –\mathrm{value in the 3rd column} \end{array}\right)/\left(\begin{array}{l} \mathrm{value in the 4th column}\\ –\mathrm{value in the 3rd column} \end{array}\right)$$

The detailed scoring algorithm for the DASH score is shown in Supplementary Table S6. A higher score represents better compliance with the DASH dietary pattern.

#### 2.2.6 Dietary acid load

Acid–base equilibrium is crucial for maintaining normal physiological function and human health. Dietary acid load (DAL) refers to the acid load from the daily diet. The potential renal acid load (PRAL) score and the net endogenous acid production (NEAP) score are the most commonly used scores to estimate DAL (31, 32). We used the following validated formulas proposed by Remer et al. (33, 34) to calculate PRAL and NEAP:

NEAP(mEq/d)=PRAL(mEq/d)+OAest(mEq/d)

$$\begin{array}{c} \mathrm{PRAL}\;\left(\mathrm{mEq}/d\right)=\left(0.49\times \mathrm{total protein}\;\left(g/\mathrm{day}\right)\right)\\ +\left(0.037\times \mathrm{phosphorus}\;\left(\mathrm{mg}/\mathrm{day}\right)\right)\\ -\left(0.021\times \mathrm{potassium}\;\left(\mathrm{mg}/\mathrm{day}\right)\right)\\ -\left(0.026\times \mathrm{magnesium}\;\left(\mathrm{mg}/\mathrm{day}\right)\right).\\ -\left(0.013\times \mathrm{calcium}\;\left(\mathrm{mg}/\mathrm{day}\right)\right) \end{array}$$

$$\mathrm{OAest}\;\left(\mathrm{mEq}/d\right)=\mathrm{Individual body surface area}\;\left(m^{2}\right)\times 41/1.73$$

Higher values of DAL indices represent higher acid load.

#### 2.2.7 Composite Dietary Antioxidant Index

The Composite Dietary Antioxidant Index (CDAI) was constructed by Wright et al. to reflect the antioxidant levels of the daily diet and then widely utilized to evaluate the association between antioxidant diets and diseases (14, 35). In this analysis, we summarized the combined intake of vitamin A, vitamin C, vitamin E, zinc, selenium, and carotenoids to calculate this antioxidant nutrient index.

$$CDAI=\sum_{i=1}^{6}\left(\frac{\left(Individualintake-Mean\right)}{SD}\right)$$

“Mean” represents the gender-specific mean intake of each nutrient; “SD” represents the gender-specific standard deviation of each nutrient. A higher CDAI represents better dietary antioxidant capacity.

#### 2.2.8 Frailty index

Frailty refers to a vulnerable state with an increased risk of adverse health outcomes, including hospitalization and mortality (36). The frailty index (FI) is an assessment instrument to quantify frailty that is well validated in previous studies (37). In this analysis, we calculated FI using a 36-item frailty index based on previous NHANES research (18). Detailed items and evaluation criteria are listed in Supplementary Table S7. Briefly, each item was evaluated and given a score between 0 and 1 to indicate the severity of the deficit. Scores for each item were summed and divided by 36 to calculate the final FI score. FI is a continuous score between 0 and 1 with higher scores representing a higher degree of frailty.

### 2.3 Statistical analysis

Basic characteristics of participants were presented for participants with CKD and all NHANES participants aged 20 years or older in 2007–2016. Continuous variables were represented as mean ± standard deviation, and categorical variables were represented as percentages. Student’s *t*-test was conducted to compare the means of dietary indices and the frailty index between the population with CKD and all participants aged ≥ 20 years in NHANES 2007–2016. Linear regression models were then used to analyze the association between each dietary score and the frailty index, with results presented as beta-coefficients with 95% confidence intervals (CIs). The mortality risk of frailty and all dietary scores was analyzed using Cox regression models and presented as hazard ratios (HR) with 95% CIs. All regression models were adjusted for basic covariates, including age, sex, ethnicity, education level, marital status, smoking status, and body mass index. Smoking status was classified as never smokers (smoked less than 100 cigarettes in life), current smokers (smoked at least 100 cigarettes in life and continued smoking now), and former smokers (smoked at least 100 cigarettes in life but quit smoking). The 8-year sampling weights were constructed based on the sampling weights provided by NHANES according to the CDC guidelines and applied to all analyses.^1^ *p* < 0.05 was considered statistically significant. All analyses were performed using R Statistical Software (v4.3.3).^2^

## 3 Results

### 3.1 Participant characteristics

A total of 4,445 participants meeting the criteria for CKD were eligible for analysis. The demographic characteristics of the study population are shown in Table 1 (characteristics of all NHANES participants aged 20 years or older in 2007–2016 were also represented as a comparison). Of the participants, 42% were men, and the mean age was 65 ± 17 years. Compared to the overall population, participants with CKD were older (65 ± 17 vs. 47 ± 17) and tended to have higher DII scores (1.61 ± 1.95 vs. 1.18 ± 2.01), NI scores (0.35 ± 0.16 vs. 0.29 ± 0.15), and FI scores (0.16 ± 0.11 vs. 0.07 ± 0.09). The CDAI scores (0.9 ± 3.6 vs. -0.4 ± 4.1), NEAP scores (54 ± 24 vs. 58 ± 27), and PRAL scores (9 ± 22 vs. 13 ± 25) of participants with CKD were lower (Table 2).

**Table 1:** Baseline characteristics of participants.

Characteristic	Population of CKD	CKD stages			Overall adults
		CKD1-2	CKD3	CKD4-5	
	*N* = 4,445	*N* = 2,048 (49%)*^1^*	*N* = 2,117 (47%)*^1^*	*N* = 257 (4%)*^1^*	*N* = 23,683
Age	65 (17)	53 (17)	73 (11)	75 (13)	47 (17)
Male	2,105 (42%)	950 (43%)	1,037 (42%)	111 (37%)	11,563 (48%)
Ethnicity					
Hispanic	927 (11%)	612 (17%)	270 (5.1%)	41 (9.5%)	6,244 (14%)
Non-Hispanic White	2,051 (68%)	792 (62%)	1,143 (76%)	104 (58%)	10,317 (68%)
Non-Hispanic Black	1,161 (14%)	445 (13%)	613 (15%)	96 (26%)	4,771 (11%)
Other ethnicities	306 (6.2%)	199 (8.7%)	91 (3.6%)	16 (6.4%)	2,351 (7.3%)
Education					
Less than high school	1,408 (23%)	662 (23%)	633 (22%)	100 (34%)	5,977 (16%)
High school	1,079 (25%)	476 (24%)	535 (25%)	64 (26%)	5,366 (22%)
Some college/associate education	1,191 (30%)	561 (31%)	559 (30%)	62 (28%)	6,901 (32%)
College Graduate or above	767 (23%)	349 (23%)	387 (23%)	29 (12%)	5,439 (29%)
Marital status					
Married	2,182 (52%)	1,000 (52%)	1,062 (53%)	114 (46%)	12,213 (54%)
Widowed	868 (17%)	239 (8.6%)	542 (24%)	74 (30%)	1,888 (5.9%)
Divorced or separated	718 (15%)	361 (16%)	314 (14%)	40 (13%)	3,419 (13%)
Never married	677 (16%)	446 (24%)	199 (8.9%)	29 (10%)	6,163 (27%)
Smoking status					
Current	766 (17%)	478 (24%)	248 (11%)	32 (10%)	4,895 (20%)
Former	1,467 (33%)	529 (27%)	831 (38%)	101 (39%)	5,780 (25%)
Never	2,212 (50%)	1,038 (50%)	1,038 (51%)	124 (51%)	13,008 (55%)
BMI group					
<18.5	83 (2.0%)	53 (3.1%)	23 (0.8%)	5 (2.2%)	368 (1.5%)
≥ 30	1,996 (44%)	952 (46%)	913 (43%)	112 (45%)	9,069 (37%)
18.5–24.9	966 (23%)	464 (24%)	437 (22%)	62 (22%)	6,358 (28%)
25.0–29.9	1,400 (31%)	579 (27%)	744 (35%)	78 (31%)	7,888 (34%)
TKCAL	1,740 (851)	1,836 (928)	1,664 (754)	1,403 (705)	1,978 (969)
DII	1.61 (1.95)	1.42 (2.02)	1.77 (1.87)	2.41 (1.70)	1.18 (2.01)
HEI-2020	52 (14)	51 (14)	52 (14)	48 (13)	51 (14)
MED	6.00 (1.23)	6.00 (1.25)	6.00 (1.21)	6.00 (1.19)	6.00 (1.25)
DASH	3.44 (1.38)	3.49 (1.37)	3.42 (1.39)	3.33 (1.48)	3.41 (1.39)
NI	0.35 (0.16)	0.32 (0.16)	0.35 (0.15)	0.48 (0.16)	0.29 (0.15)
PRAL	9 (22)	11 (24)	7 (19)	7 (19)	13 (25)
NEAP	54 (24)	55 (26)	52 (22)	50 (20)	58 (27)
CDAI	−0.9 (3.6)	−0.5 (3.8)	−1.2 (3.5)	−1.8 (2.8)	−0.4 (4.1)
FI	0.16 (0.11)	0.11 (0.10)	0.19 (0.10)	0.31 (0.10)	0.07 (0.09)
UACR	40 (677)	59 (406)	11 (551)	111 (2,268)	7 (265)
EGFR	60 (28)	93 (19)	52 (8)	22 (8)	94 (22)

^1^Median (SD); *n* (unweighted) (%).

**Table 2:** Comparison of dietary indices between the population with CKD and all participants aged ≥20 years.

Characteristics	Participants with CKD	Overall	*p* value
	*N* = 4,445	*N* = 23,683	
Age	65 (17)	47 (17)	<0.01
DII	1.61 (1.95)	1.18 (2.01)	<0.01
HEI-2020	52 (14)	51 (14)	0.5804
MED	6.00 (1.23)	6.00 (1.25)	0.6969
DASH	3.44 (1.38)	3.41 (1.39)	0.2535
NI	0.35 (0.16)	0.29 (0.15)	<0.01
PRAL	9 (22)	13 (25)	<0.01
NEAP	54 (24)	58 (27)	<0.01
CDAI	−0.9 (3.6)	−0.4 (4.1)	<0.01
FI	0.16 (0.11)	0.07 (0.09)	<0.01

Student’s t-test was used to compare the differences between the population with CKD and all participants aged ≥ 20 years in NHANES 2007–2016.

### 3.2 Association between frailty and dietary scores

**Figure 2:**
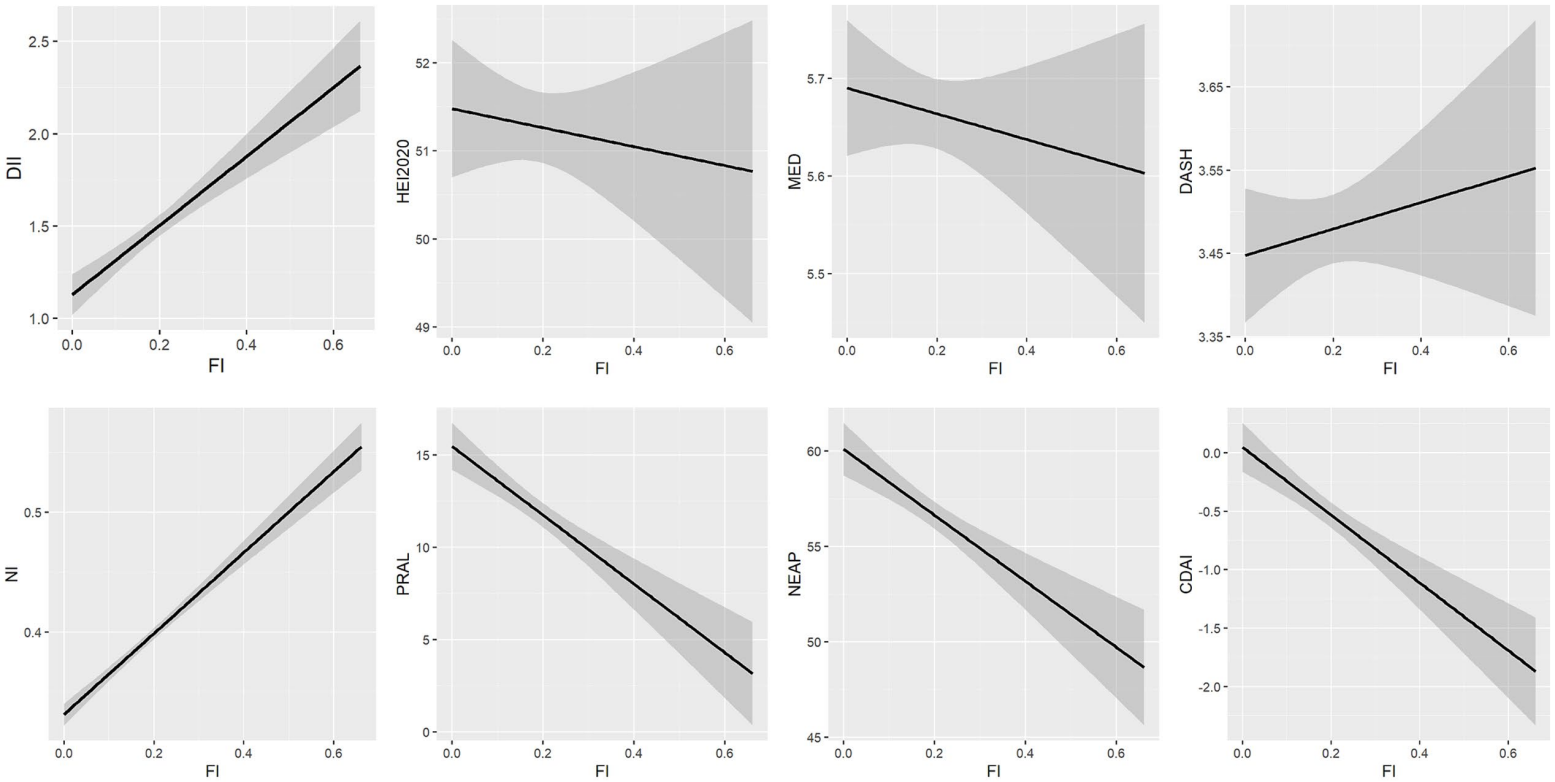
Relationship between dietary scores and frailty index.

Linear regression models were used to analyze the association between FI and each dietary score (Table 3; Figure 2). Higher DII, NI scores, and lower HEI-2020, MED, and CDAI scores were significantly associated with higher FI. PRAL, NEAP, and DASH scores were not significantly associated with frailty.

**Table 3:** Relationship between dietary scores and frailty.

Dietary scores	Unstandardized beta-coefficients (95%CI)	Standardized beta-coefficients	*p* value
DII (per 1 point)	0.00 (0.002, 0.006)	0.069	<0.001
HEI-2020 (per 1 point)	0.00 (−0.001, 0.000)	−0.063	0.002
MED (per 1 point)	−0.01 (−0.009, −0.002)	−0.058	0.003
DASH (per 1 point)	0.00 (−0.003, 0.003)	0.984	>0.9
NI (per 1 point)	0.09 (0.067, 0.122)	0.135	<0.001
PRAL (per 1 point)	0.00 (0.000, 0.000)	0.998	>0.9
NEAP (per 1 point)	0.00 (0.000, 0.000)	0.957	0.7
CDAI (per 1 point)	0.00 (−0.002, 0.000)	0.675	0.007

Linear regression models were performed to analyze the association between each dietary score and the frailty index. Models were adjusted for age, sex, ethnicity, education level, marital status, smoking status, and body mass index.

### 3.3 Association between frailty, dietary scores, and mortality

A total of 1,302 deaths were recorded by 31 December 2019: 409 deaths occurred in participants with stage 1–2 CKD, 737 deaths occurred in participants with stage 3 CKD, and 142 deaths occurred in participants with stage 4–5 CKD. Three-year, 5-year, and 8-year risk of all-cause mortality was analyzed. As indicated by Table 4, FI was associated with increased 3-year, 5-year, and 8-year mortality in populations with CKD.

**Table 4:** Relationship between frailty index and mortality.

Stage of CKD	3-year mortality		5-year mortality		8-year mortality	
	HR (95%CI)	*p* value	HR (95%CI)	*p* value	HR (95%CI)	*p* value
FI (per 0.01 point)	**1.07 (1.06, 1.08)**	**<0.001**	**1.06 (1.05, 1.07)**	**<0.001**	**1.05 (1.04, 1.06)**	**<0.001**
CKD1-2	1.06 (1.03, 1.09)	<0.001	1.05 (1.04, 1.07)	<0.001	1.05 (1.03, 1.06)	<0.001
CKD3	1.08 (1.06, 1.09)	<0.001	1.07 (1.05, 1.08)	<0.001	1.05 (1.04, 1.06)	<0.001
CKD4-5	1.04 (1.01, 1.06)	0.003	1.03 (1.01, 1.05)	<0.001	1.04 (1.02, 1.05)	0.001

A Cox regression model was used to analyze the mortality risk of frailty and all dietary scores. Models were adjusted for age, sex, ethnicity, education level, marital status, smoking status, and body mass index.

Relationship between frailty index and mortality in all individuals with CKD.

**Figure 3:**
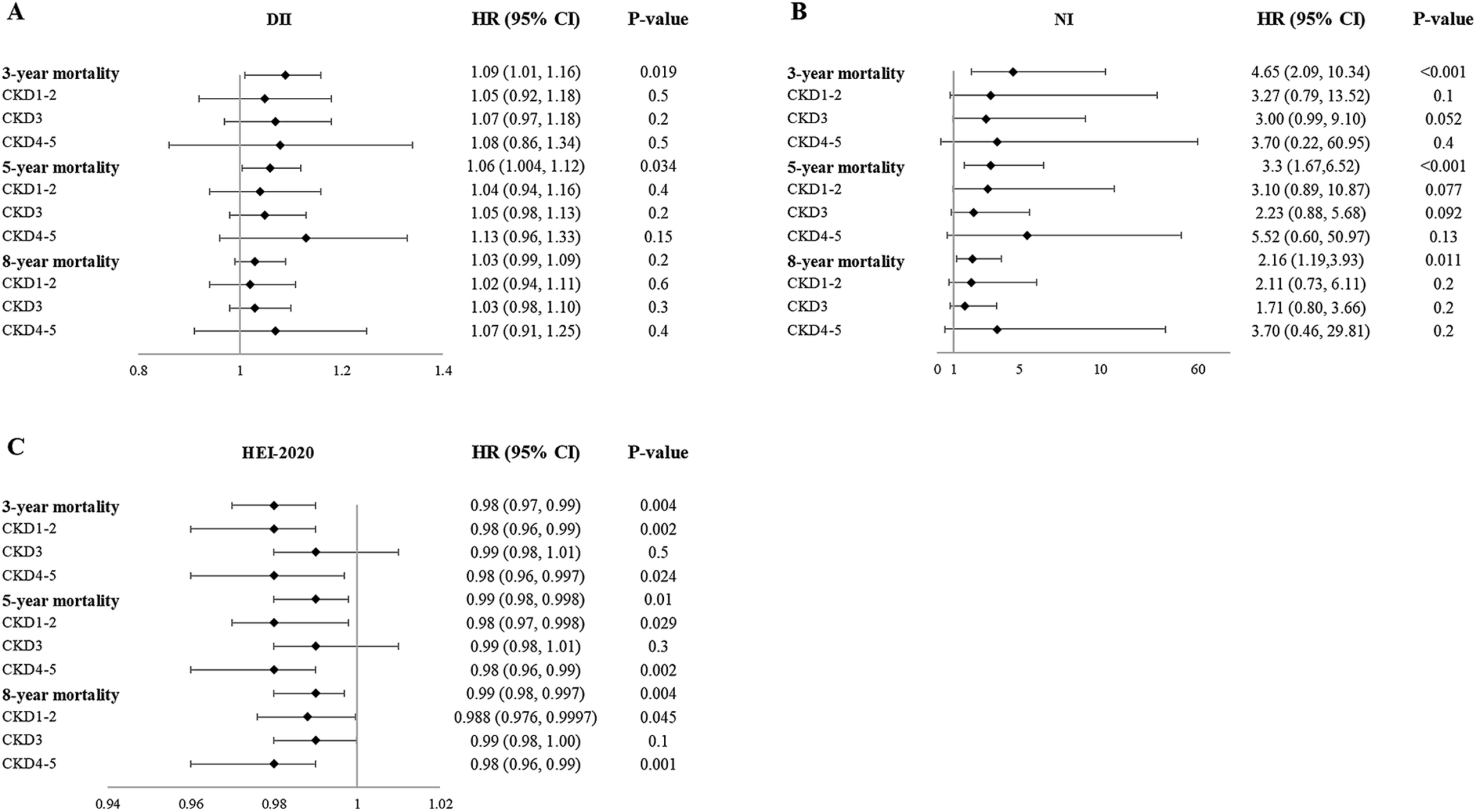
Relationship between DII **(A)**, NI **(B)**, HEI-2020 **(C)**, and mortality.

Higher DII scores, NI scores, and lower HEI-2020 scores were associated with 3-year mortality (Table 5). However, in subgroup analysis, the association between these scores and mortality varied in different CKD stages (Supplementary Table S8; Figure 3). After adjustment for FI, DII scores were not significantly associated with 3-year mortality, while the mortality risk of higher NI and lower HEI-2020 scores still remained.

**Table 5:** Relationship between dietary scores and mortality.

Dietary scores	3-year mortality		5-year mortality		8-year mortality	
	HR (95%CI)	*p* value	HR (95%CI)	*p* value	HR (95%CI)	*p* value
Model 1: Adjusted for basic covariates^1^						
DII (per 1 point)	1.09 (1.01, 1.16)	0.019	1.06 (1.004, 1.12)	0.034	1.03 (0.99, 1.09)	0.2
NI (per 1 point)	4.65 (2.09, 10.34)	<0.001	3.3 (1.67,6.52)	<0.001	2.16 (1.19,3.93)	0.011
HEI-2020 (per 1 point)	0.98 (0.97, 0.99)	0.004	0.99 (0.98, 0.998)	0.01	0.99 (0.98, 0.997)	0.004
MDS (per 1 point)	0.9 (0.84, 1.04)	0.2	0.94 (0.87, 1.02)	0.15	0.93 (0.87, 1.00)	0.051
DASH (per 1 point)	0.95 (0.86, 1.05)	0.4	0.95 (0.88, 1.02)	0.2	0.96 (0.90, 1.02)	0.2
PRAL (per 1 point)	1.00 (0.996, 1.01)	0.8	1.00 (0.998, 1.01)	0.3	1.00 (0.999, 1.01)	0.2
NEAP (per 1 point)	0.999 (0.99, 1.00)	0.8	1.01 (1.00, 1.02)	0.6	1.00 (0.998, 1.01)	0.4
CDAI (per 1 point)	0.99 (0.96, 1.03)	0.8	1.00 (0.97, 1.03)	0.9	1.00 (0.97, 1.02)	0.8
Model 2: Adjusted for basic covariates + FI						
DII (per 1 point)	1.06 (0.99, 1.12)	0.094	1.04 (0.99, 1.09)	0.13	1.02 (0.97, 1.07)	0.3
NI (per 0.1 point)	2.41 (1.12, 5.22)	0.025	1.96 (1.04, 3.70)	0.037	1.47 (0.84, 2.55)	0.2
HEI-2020 (per 1 point)	0.987 (0.977, 0.998)	0.024	0.99 (0.98, 1.00)	0.057	0.99 (0.98, 0.999)	0.02
MDS (per 1 point)	0.98 (0.88, 1.08)	0.6	0.97 (0.90, 1.05)	0.5	0.96 (0.89, 1.03)	0.2
DASH (per 1 point)	0.95 (0.85, 1.05)	0.3	0.94 (0.88, 1.02)	0.14	0.95 (0.90, 1.01)	0.11
PRAL (per 1 point)	1.00 (0.997, 1.01)	0.5	1.00 (0.998, 1.01)	0.2	1.00 (0.9993, 1.01)	0.1
NEAP (per 1 point)	1.00 (0.996, 1.01)	0.8	1.00 (0.997, 1.01)	0.4	1.00 (0.999, 1.01)	0.2
CDAI (per 1 point)	1.00 (0.97, 1.04)	0.8	1.00 (0.98,1.03)	0.7	1.00 (0.98, 1.03)	>0.9

Multivariable-adjusted Cox regression analysis was used to analyze the mortality risk of frailty and all dietary scores. ^1^Basic covariates: age, sex, ethnicity, education level, marital status, smoking status, and body mass index.

Similarly, higher DII scores, NI scores, and lower HEI-2020 scores were associated with 5-year mortality. After adjustment for FI, DII, and HEI-2020 scores were not related to 5-year mortality, while the mortality risk of higher NI scores still remained (Table 5).

Regarding the 8-year mortality, higher NI scores and lower HEI-2020 scores were associated with 8-year mortality. After adjustment for FI, NI scores were not related to 8-year mortality, while the mortality risk of lower HEI-2020 scores still remained (Table 5). These results demonstrated that short- and medium-term mortality was more influenced by current nutritional/inflammatory status (NI, DII), while long-term mortality was more influenced by sustained lifestyle quality (HEI-2020).

MED, DASH, PRAL, NEAP, and CDAI scores were not significantly associated with mortality in the overall population of CKD, and this relationship remained after adjustment for FI (Table 5). In the subgroup analysis, DASH (3-year, 5-year, and 8-year), PRAL (8-year), and NEAP (8-year) scores were related to increased mortality risk in individuals with stage 4–5 CKD. Consequently, in individuals with stage 4–5 CKD, emphasis should be placed on balancing individualized potassium/acid restriction with adequate energy and protein intake, rather than simply emphasizing higher fruit and vegetable intake. The mortality risk of dietary scores in the population at different stages of CKD is listed in Supplementary Table S8.

## 4 Discussion

This cross-sectional study collected data from NHANES (2007–2016) and analyzed the association between FI and dietary indices as well as their mortality risk among adults with CKD. Our results revealed the association between higher DII, NI scores, and lower HEI-2020, MED, and CDAI scores with higher frailty risk. In multivariate Cox regression analysis, higher DII scores, NI scores, and lower HEI-2020 scores were associated with 3-year, 5-year, and 8-year all-cause mortality in individuals with CKD. However, when controlling for FI, only higher NI scores (3-year and 5-year mortality) and lower HEI-2020 (3-year and 8-year mortality) scores were related to higher mortality risk.

A recent survey utilizing data from NHANES found that NI, Energy-Adjusted Dietary Inflammatory Index (E-DII), HEI-2015, MED, and DASH scores are associated with frailty and 8-year mortality risk in adults across all ages (18). In our study, only DII scores, NI scores, and HEI-2020 were associated with mortality in CKD patients. These differences may be attributed to a different study population.

DII is an index assessing the inflammatory potential of diets, and chronic systemic inflammation is an important characteristic of CKD, which could contribute to the dysfunction and fibrosis of the kidney, thus precipitating the progression of CKD (38). Higher levels of DII significantly increase the risk of CKD both in middle-aged (40–59) and elderly (≥ 60) populations (39). Higher DII scores were also associated with an increased risk of cognitive impairment and increased all-cause mortality in individuals with CKD. These were consistent with our results (40, 41).

Among these dietary indices, the NI score and HEI are more comprehensive than the others. Except for fruits and vegetables, HEI components also include sodium and sugar intake. Fluid and sodium retention play an important role in the pathophysiology of CKD, and restriction of sodium intake is recommended by KDIGO (24). A recent study suggested that consumption of sugar-sweetened or artificially sweetened beverages was associated with the risk of developing CKD (42). NI is a more comprehensive dietary score that includes nutrient intakes, anthropometric measurements, and blood tests. Since risk factors for the development and progression of CKD are very complex, more comprehensive indices may be more reliable for the assessment of prognosis.

Composite dietary antioxidant index (CDAI) was constructed to reflect the antioxidant levels of the daily diet (14, 35). Our analyses indicated that CDAI was related to frailty but not to mortality risk in the CKD population. Research on the association between CDAI and mortality risk in the CKD population is very limited. Li et al. (43) included participants in NHANES 2007–2018 and reported that no associations were observed between CDAI and all-cause mortality among participants with CKD stages 3–5. However, another study including participants in NHANES 2001–2018 found that CDAI was related to all-cause mortality in adults with CKD (44). We noticed that both studies only broadly investigated the mortality rates without specifically investigating the 3-year, 5-year, and 8-year mortality rates. Consequently, the mortality risk of CDAI in CKD remains to be further explored.

In our analysis, MED and DASH scores were not associated with increased mortality risk in CKD. The results from another survey in NHANES revealed that the DASH diet was associated with a lower risk of end-stage kidney disease but was not associated with mortality (45), which was in accordance with our results. Kalani et al. (46) summarized evidence about the effects of the DASH diet in CKD and expressed concerns about recommending the DASH diet to populations with CKD, especially those in stage 5. They proposed that more reliable evidence is necessary to clarify the effects of the DASH diet in individuals with CKD. Xiaoyan et al. (47) categorized the adherence to the MED diet as low, medium, and high adherence according to the scores and analyzed the relationship between the MED diet and mortality. The results suggested that better adherence to the MED diet predicted 10-year survival in populations with CKD. A similar conclusion was demonstrated by a prospective cohort study (48). Reasons for inconsistency with our results may lie in different analytical methods, as they adopted a trend test that is usually used to demonstrate a dose–response effect between the exposure and outcomes (49). Further research is required to assess the mortality risk of the MED diet in individuals with CKD. We noted that adequate intake of potassium is one of the components included in the DASH diet. However, hyperkalemia often occurred in patients with CKD and was related to a higher risk of end-stage renal disease and mortality (50).

Acid–base balance disturbance is an important characteristic of CKD and is considered a risk factor for the progression of CKD. However, we found that compared to the general population, NEAP and PRAL were lower in participants with CKD. This is probably because the age of participants with CKD was significantly higher. In a study assessing the impact of DAL on cognitive functions, the NEAP and PRAL of individuals over 80 years were significantly lower than those of individuals aged 60–69 (16). In our analysis, there was no association between PRAL scores, NEAP scores, and all-cause mortality. These results aligned with previous studies (51, 52). Tanushree et al. (52) indicated that higher levels of DAL were associated with increased risk of ESRD but not associated with increased risk of mortality. A cohort study including 442 patients with CKD also revealed that neither NEAP nor PRAL was associated with mortality (51). Consequently, higher DAL may impact kidney function and accelerate the progression of CKD, and dietary prescriptions for patients with advanced CKD must balance acid load and high potassium risk management. However, the relationship between DAL and CKD mortality remains unclear.

Our analysis suggested that the relationship between DII score, NI score, HEI-2020 score, and all-cause mortality altered after adjustment for frailty: NI score was not related to mortality, higher NI scores were only related to 3-year and 5-year mortality, and lower HEI-2020 scores were only associated with 3-year and 8-year mortality. These results indicate that frailty could impact the association between dietary indices and mortality risk in CKD, which was in accordance with previous studies in the adult population (18).

However, several limitations exist in our study. First, this analysis was cross-sectional, and prospective studies are needed to validate these conclusions further. Furthermore, the diagnostic criteria of CKD in our study were based on serum creatinine and urine albumin creatinine ratio, instead of longitudinal observation of kidney structure and function for 3 months. In addition, decreased appetite, inflammation, and frailty themselves could influence dietary intake, leading to causality bias. However, we noted that even after adjusting for FI, NI, and HEI-2020 retained their independent predictive value. Finally, many participants were excluded from the analysis because of missing data, which may bring selection bias.

## 5 Conclusion

In conclusion, our analysis revealed that in adults with CKD, the FI was higher compared to the general population and associated with DII, NI, CDAI, HEI-2020, and MED scores. Regardless of the dietary index used, the diet for individuals with CKD was not ideal, which could lead to frailty. FI was significantly associated with increased all-cause mortality risk in populations with CKD. Higher DII scores, NI scores, and lower HEI-2020 scores were related to mortality risk. However, only higher NI scores (3-year and 5-year mortality) and lower HEI-2020 (3-year and 8-year mortality) scores were related to higher mortality risk after adjustment for FI. These results indicated that dietary interventions and management of frailty could improve the prognosis in populations with CKD. DII, NI, and HEI-2020 could be better prognostic factors for CKD patients than the remaining four indices.

## Data Availability

The original contributions presented in the study are included in the article/Supplementary material, further inquiries can be directed to the corresponding authors.

## Footnotes

^1^ https://wwwn.cdc.gov/nchs/nhanes/tutorials/

^2^ https://www.R-project.org/
