# Supplementary material for: Relationship between different diet indices and frailty and mortality in population with CKD
Source: Front Nutr. 2025 Oct 7;12:1602587. doi: 10.3389/fnut.2025.1602587 (PMC12537386; doi:10.3389/fnut.2025.1602587)
Supplement: Supplementary file 1 [file Table_1.DOCX]

Supplementary Material

**Supplementary Table S1. Dietary related variables included in calculations of the dietary scores.**

|  | **NI** | **DII** | **HEI-2020** | **MED** | **DASH** | **DAL** | **CDAI** |
| --- | --- | --- | --- | --- | --- | --- | --- |
| Energy | x | x | x |  | x |  |  |
| Protein | x | x |  |  | x | x |  |
| Carbohydrate | x | x |  |  |  |  |  |
| Total fatty acids |  | x |  |  | x |  |  |
| Saturated fatty acids | x | x | x | x | x |  |  |
| MUFAs |  | x | x | x |  |  |  |
| PUFAs |  | x | x |  |  |  |  |
| Omega-3 fatty acids |  | x |  |  |  |  |  |
| Omega-6 fatty acids |  | x |  |  |  |  |  |
| EPA and DHA | x |  |  |  |  |  |  |
| Cholesterol |  | x |  |  | x |  |  |
| Fiber |  | x |  |  | x |  | x |
| Vitamin A | x | x |  |  |  |  |  |
| Vitamin B1 | x | x |  |  |  |  |  |
| Vitamin B2 | x | x |  |  |  |  |  |
| Vitamin B3 | x | x |  |  |  |  |  |
| Vitamin B6 | x | x |  |  |  |  |  |
| Vitamin B12 |  | x |  |  |  |  |  |
| Folate | x | x |  |  |  |  |  |
| Vitamin C | x | x |  |  |  |  | x |
| Vitamin D |  | x |  |  |  |  |  |
| Vitamin E |  | x |  |  |  |  | x |
| Beta-carotene |  | x |  |  |  |  | x |
| Alpha-carotene |  |  |  |  |  |  | x |
| Beta-cryptoxanthin |  |  |  |  |  |  | x |
| Lycopene |  |  |  |  |  |  | x |
| Lutein and zeaxanthin |  |  |  |  |  |  | x |
| Calcium |  |  |  |  | x | x |  |
| Copper | x |  |  |  |  |  |  |
| Iron |  | x |  |  |  |  |  |
| Magnesium |  | x |  |  | x | x |  |
| Phosphorus | x |  |  |  |  | x |  |
| Potassium |  |  |  |  | x | x |  |
| Selenium | x | x |  |  |  |  | x |
| Sodium | x |  | x |  | x |  |  |
| Zinc |  | x |  |  |  |  | x |
| Alcohol |  | x |  | x |  |  |  |
| Caffeine |  | x |  |  |  |  |  |
| Dairy product |  |  | x | x |  |  |  |
| Whole grains |  |  | x | x |  |  |  |
| Refined grains |  |  | x | x |  |  |  |
| Fruit juice |  |  | x |  |  |  |  |
| Whole fruits |  |  | x | x |  |  |  |
| Nuts |  |  |  | x |  |  |  |
| Total vegetables |  |  | x | x |  |  |  |
| Greens and beans |  |  | x |  |  |  |  |
| Red meat and product |  |  | x | x |  |  |  |
| Poultry |  |  | x | x |  |  |  |
| Seafood |  |  | x | x |  |  |  |
| Plant proteins |  |  | x |  |  |  |  |
| Added sugars |  |  | x |  |  |  |  |

NI, Nutrition Index; DII, Dietary inflammatory index; HEI-2020, Healthy eating index-2020; MED, Mediterranean diet score; DASH, Dietary approaches to stop hypertension; DAL, Dietary acid load; CDAI, Composite dietary antioxidant index; MUFAs, Monounsaturated fatty acids; PUFAs, Polyunsaturated fatty acids; EPA, eicosapentaenoic acid; DHA, docosahexaenoic acid;

**Supplementary Table S2. Scoring algorithm for Nutrition Index.**

| **Components** | **Normal range** | **0 point** | **1 point** |
| --- | --- | --- | --- |
| **Nutrient intakes** | | | |
| Energy (kcal/day) | M ≥ 2400, F ≥ 1800 | Normal range | M < 2400, F < 1800 |
| Energy per weight  (kcal/kg/day) | 25-35 | ≥ 25 | < 25 |
| Protein (g/day) | M ≥ 56, F ≥ 46 | Normal range | M < 56, F < 46 |
| Protein per weight (g/kg/day) | < 65 years, ≥ 0.8 | Normal range | < 65 years, < 0.8 |
|  | ≥ 65 years, ≥ 1 |  | ≥ 65 years, < 1.0 |
| Carbohydrate (g/day) | ≥ 180 | Normal range | < 180 |
| Percentage of saturated fat (%) | < 10 | Normal range | ≥ 10 |
| Vitamin A, RAE (mcg/day) | M 900-3000 | Normal range | M < 900 or > 3000 |
|  | F 700-3000 |  | F < 700 or > 3000 |
| Vitamin C (mg/day) | M 90-2000,  F 75-2000 | Normal range | M < 90 or > 2000 |
|  |  |  | F < 75 or > 2000 |
| Thiamin (mg/day) | M ≥ 1.2, F ≥ 1.1 | Normal range | M < 1.2, F < 1.1 |
| Riboflavin (mg/day) | M ≥ 1.3, F ≥ 1.1 | Normal range | M < 1.3, F < 1.1 |
| Niacin (mg/day) | M 16-35, F 14-35 | Normal range | M < 16 or > 35 |
|  |  |  | F < 14 or > 35 |
| Pyridoxine (mg/day) | ≤ 50 years, 1.3-100 | Normal range | ≤ 50 years,  < 1.3 or > 100 |
|  | > 50 years, M 1.7-100 |  | > 50 years,  M < 1.7 or > 100 |
|  | > 50 years, F 1.5-100 |  | > 50 years,  F < 1.5 or > 100 |
| Folate (mcg/day) | 400-1000 | Normal range | < 400 or > 1000 |
| Phosphorous (mg/day) | 700-4000 | Normal range | < 700 or > 4000 |
| Copper (mg/day) | 0.9-10 | Normal range | < 0.9 or > 10 |
| Sodium (mg/day) | ≤ 50 years,  1500-2300 | ≤ 50 years, ≥1,500 | ≤ 50 years, < 1500 |
|  | > 50-70 years,  1300-2300 | > 50-70 years, ≥ 1300 | > 50-70 years, < 1300 |
|  | > 70 years,  1200-2300 | > 70 years, ≥1200 | > 70 years, < 1200 |
| Selenium (mcg/day) | 55-400 | Normal range | < 55 or > 400 |
| Fish oil (g/day) | ≥ 0.25 | Normal range | < 0.25 |
| **Anthropometric measurements** | | | |
| Body mass index (kg/m2) | 18.5-24.9 | 18.5-29.9 | < 18.5 or ≥ 30.0 |
| Body weight change in  past 1 year (%) | ≤ 10 | Normal range | > 10 |
| Waist circumference (cm) | M < 94, F < 80 | Normal range | M ≥ 94, F ≥ 80 |
| **Blood tests** | | | |
| Total lymphocyte count  (cells/mm3) | > 1500 | Normal range | ≤ 1500 |
| Hemoglobin (g/dL) | M 13.5-18.0 | Normal range | M < 13.5 or > 18.0 |
|  | F 12.0-16.0 |  | F < 12.0 or > 16.0 |
| Mean corpuscular volume (fL) | 80-100 | Normal range | < 80 or > 100 |
| Albumin (g/L) | 35-55 | Normal range | < 35 or > 55 |
| Vitamin D (ng/mL) | 20-50 | Normal range | < 20 or > 50 |
| Iron, serum (mcg/dL) | 50-180 | Normal range | < 50 or > 180 |
| Creatinine (mg/dL) | M 0.80–1.40, | Normal range | M < 0.80 or > 1.40 |
|  | F 0.56–1.00 |  | F < 0.56 or > 1.00 |
| Triglyceride (mg/dL) | < 150 | Normal range | ≥ 150 |
| HDL-c (mg/dL) | M > 40, F > 50 | Normal range | M ≤ 40, F ≤ 50 |
| Glucose (mg/dL) | 70-100 | Normal range | < 70 or > 100 |

**Supplementary Table S3. Calculation for Dietary inflammatory index.**

| **Food parameter** | **Unit (per day)** | **Overall inflammatory**  **effect score** | **Global daily mean**  **intake (units/d)** | **SD** |
| --- | --- | --- | --- | --- |
| Alcohol | g | -0.278 | 13.98 | 3.72 |
| vitamin B12 | μg | 0.106 | 5.15 | 2.7 |
| vitamin B6 | mg | -0.365 | 1.47 | 0.74 |
| Beta-carotene | μg | -0.584 | 3718 | 1720 |
| Caffeine | g | -0.11 | 8.05 | 6.67 |
| Carbohydrate | g | 0.097 | 272.2 | 40 |
| Cholesterol | mg | 0.11 | 279.4 | 51.2 |
| Energy | kcal | 0.18 | 2056 | 338 |
| Total fat | g | 0.298 | 71.4 | 19.4 |
| Fiber | g | -0.663 | 18.8 | 4.9 |
| Folic acid | μg | -0.19 | 273 | 70.7 |
| Iron | mg | 0.032 | 13.35 | 3.71 |
| Magnesium | mg | -0.484 | 310.1 | 139.4 |
| MUFA | g | -0.009 | 27 | 6.1 |
| Niacin | mg | -0.246 | 25.9 | 11.77 |
| n-3 fatty acid | g | -0.436 | 1.06 | 1.06 |
| n-6 fatty acid | g | -0.159 | 10.8 | 7.5 |
| Protein | g | 0.021 | 79.4 | 13.9 |
| PUFA | g | -0.337 | 13.88 | 3.76 |
| Riboflavin | mg | -0.068 | 1.7 | 0.79 |
| Saturated fat | g | 0.373 | 28.6 | 8 |
| Selenium | μg | -0.191 | 67 | 25.1 |
| Thiamin | mg | -0.098 | 1.7 | 0.66 |
| Vitamin A | RE | -0.401 | 983.9 | 518.6 |
| Vitamin C | mg | -0.424 | 118.2 | 43.46 |
| Vitamin D | μg | -0.446 | 6.26 | 2.21 |
| Vitamin E | mg | -0.419 | 8.73 | 1.49 |
| Zinc | mg | -0.313 | 9.84 | 2.19 |

MUFA, Monounsaturated fatty acids; PUFA, Polyunsaturated fatty acids.

**Supplementary Table S4. Scoring algorithm for Healthy eating index-2020.**

| **Components** | **Max Score** | **Standard for**  **maximum score** | **Standard for minimum**  **score of 0** |
| --- | --- | --- | --- |
| **Adequacy** |  |  |  |
| Total fruit | 5 | ≥0.8 cup eq. per 1,000 kcal | No Fruit |
| Whole fruit | 5 | ≥0.4 cup eq. per 1,000 kcal | No Whole Fruit |
| Total vegetable | 5 | ≥1.1 cup eq. per 1,000 kcal | No Vegetables |
| Greens and beans | 5 | ≥0.2 cup eq. per 1,000 kcal | No Dark Green Vegetables or Legumes |
| Total protein food | 5 | ≥2.5 oz eq. per 1,000 kcal | No Protein Foods |
| Seafood and plant  protein | 5 | ≥0.8 oz eq. per 1,000 kcal | No Seafood or Plant Proteins |
| Whole grain | 10 | ≥1.5 oz eq. per 1,000 kcal | No Whole Grains |
| Dairy | 10 | ≥1.3 cup eq. per 1,000 kcal | No Dairy |
| Fatty Acids | 10 | ≥2.5 | ≤1.2 |
| **Moderation** |  |  |  |
| Refined grain | 10 | ≤1.8 oz eq. per 1,000 kcal | ≥4.3 oz eq. per 1,000 kcal |
| Sodium | 10 | ≤1.1 g per 1,000 kcal | ≥2.0 g per 1,000 kcal |
| Added sugar | 10 | ≤6.5% of energy | ≥26% of energy |
| Saturated fat | 10 | ≤8% of energy | ≥16% of energy |

**Supplementary Table S5. Scoring algorithm for Mediterranean Diet Score.**

| **Food Groups** | **Minimum score: 0** | **Maximum score: 1** |
| --- | --- | --- |
| Whole Fruit + Fruit juice | < Median | ≥ Median |
| Vegetable | < Median | ≥ Median |
| Whole grain product | < Median | ≥ Median |
| Legume | < Median | ≥ Median |
| Nut | < Median | ≥ Median |
| Fish | < Median | ≥ Median |
| Red and processed meat | ≥ Median | < Median |
| Ratio of Monounsaturated lipid  to saturated lipid | < Median | ≥ Median |
| Alcohol | < 10 or >25 g/d | 10-25 g/day |

**Supplementary Table S6. Scoring algorithm for Dietary Approaches to Stop Hypertension Score.**

| **Components** | **Units** | **Minimum score: 0** | **Maximum score: 1** |
| --- | --- | --- | --- |
| Total fat | % of total kcal | ≥ 37 | ≤ 27 |
| Saturated fat | % of total kcal | ≥ 16 | ≤ 6 |
| Protein | % of total kcal | ≤ 15 | ≥ 18 |
| Cholesterol | mg/day per 2000 kcal | ≥ 285.7 | ≤ 142.8 |
| Fiber | g/day per 2000 kcal | ≤ 8.6 | ≥ 29.5 |
| Potassium | mg/day per 2000 kcal | ≤ 1619 | ≥ 4476 |
| Magnesium | mg/day per 2000 kcal | ≤ 157 | ≥ 476 |
| Calcium | mg/day per 2000 kcal | ≤ 429 | ≥ 1181 |
| Sodium | mg/day per 2000 kcal | ≥ 2857 | ≤ 2286 |

**Supplementary Table S7. 36-item Frailty Index.**

| **Self-reported Frailty Index items** |  |
| --- | --- |
| 1. Angina/angina pectoris | 14. Difficulty lifting or carrying |
| 2. Heart attack | 15. Difficulty walking between rooms on same floor |
| 3. Coronary heart disease | 16. Difficulty standing up from an armless chair |
| 4. Stroke | 17. Difficulty getting in and out of bed |
| 5. Thyroid condition | 18. Difficulty dressing yourself |
| 6. Cancer | 19. Difficulty grasping/holding small objects |
| 7. Arthritis | 20. Difficulty attending social events |
| 8. High blood pressure | 21. Self-reported health |
| 9. Diabetes mellitus | 22. Frequency of healthcare use |
| 10. Weak/failing kidneys | 23. Health compared to 1 year ago |
| 11. Confusion or inability to remember things | 24. Overnight hospital stays |
| 12. Difficulty managing money | 25. Medications |
| 13. Difficulty stooping, crouching, kneeling |  |
| **Laboratory Frailty Index items** |  |
| 26. Pulse rate (60-99 bpm) | 32. Red cell distribution width (≤14.6%) |
| 27. Systolic blood pressure (90-140 mmHg) | 33. Lactate dehydrogenase (≤190 U/L) |
| 28. Pulse pressure (30-60 mmHg) | 34. Alkaline phosphatase (≤115 U/L) |
| 1. Platelet count SI   (150- 450 unit 1000 cells/μL) | 35. Uric acid (M: 240-510, F: 160-430 umol/L) |
| 30. Blood urea nitrogen (3-20 mg/dL) | 36. Total calcium (2.0-2.5 mmol/L) |
| 31. Bicarbonate (≤28 mmol/L) |  |

**Supplementary Table S8. Subgroup analysis of relationship between dietary scores and mortality in different stages of CKD.**

| **Dietary scores** | **3-year mortality** | | **5-year mortality** | | **8-year mortality** | |
| --- | --- | --- | --- | --- | --- | --- |
|  | **HR (95%CI)** | **p value** | **HR (95%CI)** | **p value** | **HR (95%CI)** | **p value** |
| **DII (per 1 point)** | **1.09 (1.01, 1.16)** | **0.019** | **1.06 (1.004, 1.12)** | **0.034** | **1.03 (0.99, 1.09)** | **0.2** |
| CKD1-2 | 1.05 (0.92, 1.18) | 0.5 | 1.04 (0.94, 1.16) | 0.4 | 1.02 (0.94, 1.11) | 0.6 |
| CKD3 | 1.07 (0.97, 1.18) | 0.2 | 1.05 (0.98, 1.13) | 0.2 | 1.03 (0.98, 1.10) | 0.3 |
| CKD4-5 | 1.08 (0.86, 1.34) | 0.5 | 1.13 (0.96, 1.33) | 0.15 | 1.07 (0.91, 1.25) | 0.4 |
| **NI (per 1 point)** | **4.65 (2.09, 10.34)** | **<0.001** | **3.3 (1.67,6.52)** | **<0.001** | **2.16 (1.19,3.93)** | **0.011** |
| CKD1-2 | 3.27 (0.79, 13.52) | 0.1 | 3.10 (0.89, 10.87) | 0.077 | 2.11 (0.73, 6.11) | 0.2 |
| CKD3 | 3.00 (0.99, 9.10) | 0.052 | 2.23 (0.88, 5.68) | 0.092 | 1.71 (0.80, 3.66) | 0.2 |
| CKD4-5 | 3.70 (0.22, 60.95) | 0.4 | 5.52 (0.60, 50.97) | 0.13 | 3.70 (0.46, 29.81) | 0.2 |
| **HEI-2020 (per 1 points)** | **0.98 (0.97, 0.99)** | **0.004** | **0.99 (0.98, 0.998)** | **0.01** | **0.99 (0.98, 0.997)** | **0.004** |
| CKD1-2 | 0.98 (0.96, 0.99) | 0.002 | 0.98 (0.97, 0.998) | 0.029 | 0.988 (0.976, 0.9997) | 0.045 |
| CKD3 | 0.99 (0.98, 1.01) | 0.5 | 0.99 (0.98, 1.01) | 0.3 | 0.99 (0.98, 1.00) | 0.10 |
| CKD4-5 | 0.98 (0.96, 0.997) | 0.024 | 0.98 (0.96, 0.99) | 0.002 | 0.98 (0.96, 0.99) | 0.001 |
| **MDS (per 1 point)** | **0.9 (0.84, 1.04)** | **0.2** | **0.94 (0.87, 1.02)** | **0.15** | **0.93 (0.87, 1.00)** | **0.051** |
| CKD1-2 | 0.97 (0.82, 1.15) | 0.7 | 0.90 (0.78, 1.03) | 0.11 | 0.91 (0.81, 1.02) | 0.1 |
| CKD3 | 1.00 (0.89, 1.14) | >0.9 | 1.00 (0.91, 1.11) | >0.9 | 0.97 (0.89, 1.05) | 0.4 |
| CKD4-5 | 0.84 (0.67, 1.06) | 0.13 | 0.86 (0.72, 1.04) | 0.12 | 0.88 (0.73, 1.06) | 0.2 |
| **DASH (per 1 point)** | **0.95 (0.86, 1.05)** | **0.4** | **0.95 (0.88, 1.02)** | **0.2** | **0.96 (0.90, 1.02)** | **0.2** |
| CKD1-2 | 0.92 (0.82, 1.03) | 0.13 | 0.89 (0.80, 1.01) | 0.082 | 0.98 (0.88, 1.09) | 0.7 |
| CKD3 | 1.05 (0.90, 1.22) | 0.5 | 1.00 (0.90, 1.11) | >0.9 | 0.97 (0.90, 1.05) | 0.5 |
| CKD4-5 | 0.82 (0.68, 0.999) | 0.048 | 0.82 (0.70, 0.97) | 0.017 | 0.82 (0.71, 0.94) | 0.006 |
| **PRAL (per 1 point)** | **1.00 (0.996, 1.01)** | **0.8** | **1.00 (0.998, 1.01)** | **0.3** | **1.00 (0.999, 1.01)** | **0.2** |
| CKD1-2 | 1.00 (0.99, 1.01) | 0.7 | 1.01 (0.9997, 1.01) | 0.062 | 1.00 (0.997, 1.01) | 0.3 |
| CKD3 | 1.00 (0.99, 1.01) | 0.7 | 1.00 (0.99, 1.01) | 0.9 | 1.00 (0.996, 1.01) | 0.7 |
| CKD4-5 | 1.01 (1.00, 1.02) | 0.2 | 1.01 (0.997, 1.02) | 0.2 | 1.01 (1.00, 1.02) | 0.035 |
| **NEAP (per 1 point)** | **0.999 (0.99, 1.00)** | **0.8** | **1.01 (1.00, 1.02)** | **0.6** | **1.00 (0.998, 1.01)** | **0.4** |
| CKD1-2 | 1.00 (0.99, 1.01) | >0.9 | 1.006 (0.998, 1.01) | 0.13 | 1.00 (0.997, 1.01) | 0.3 |
| CKD3 | 1.00 (0.99, 1.01) | 0.5 | 0.998 (0.99, 1.00) | 0.5 | 1.00 (0.99, 1.00) | 0.9 |
| CKD4-5 | 1.01 (0.99, 1.02) | 0.3 | 1.01 (0.996, 1.02) | 0.2 | 1.01 (1.00, 1.02) | 0.049 |
| **CDAI (per 1 point)** | **0.99 (0.96, 1.03)** | **0.8** | **1.00 (0.97, 1.03)** | **0.9** | **1.00 (0.97, 1.02)** | **0.8** |
| CKD1-2 | 1.02 (0.96, 1.09) | 0.5 | 1.00 (0.95, 1.06) | 0.9 | 1.00 (0.96, 1.05) | >0.9 |
| CKD3 | 0.99 (0.94, 1.04) | 0.6 | 1.00 (0.96, 1.04) | 0.9 | 0.99 (0.96, 1.03) | 0.8 |
| CKD4-5 | 1.04 (0.95, 1.14) | 0.4 | 1.00 (0.92, 1.09) | >0.9 | 1.03 (0.97, 1.10) | 0.3 |

**Adjusted by basic covariates:** age, sex, race, education level, marital status, smoking status and body mass index.
